# Surgical Treatment of Obesity in Latinos and African Americans: Future Directions and Recommendations to Reduce Disparities in Bariatric Surgery

**DOI:** 10.1089/bari.2017.0037

**Published:** 2018-03-01

**Authors:** Emily Daviau Smith, Brian T. Layden, Chandra Hassan, Lisa Sanchez-Johnsen

**Affiliations:** ^1^ Division of Endocrinology, Diabetes, and Metabolism, Department of Medicine, University of Illinois at Chicago, Chicago, Illinois.; ^2^ Jesse Brown Veterans Affairs Medical Center, Chicago, Illinois.; ^3^ Department of Surgery, University of Illinois at Chicago, Chicago, Illinois.; ^4^ Department of Psychiatry, University of Illinois at Chicago, Chicago, Illinois.

**Keywords:** disparities, Hispanics/Latinos, African Americans/blacks

## Abstract

***Introduction:*** Obesity and metabolic syndrome are increasingly prevalent in the United States, particularly among African Americans and Latinos. Bariatric surgery has become one of the primary treatment modalities for obesity and type 2 diabetes. However, fewer Latinos and African Americans are undergoing bariatric surgery than whites. The aim of this article is to describe the disparities in seeking and accessing bariatric surgery, describe the outcomes following bariatric procedures in Latinos and African Americans, and offer recommendations and future research directions that may assist in addressing these disparities.

***Methods:*** Original research and review articles published in English were reviewed.

***Results:*** Potential reasons why Latinos and African Americans have low rates of seeking bariatric surgery are described. Disparities in access to care and financial coverage, low rates of referral by primary care providers, and cultural attitudes toward obesity in conjunction with mistrust of the healthcare system are discussed as potential contributors to the low rate of bariatric surgery in Latinos and African Americans. Finally, disparities in bariatric surgery outcomes, comorbidities, and complications are reviewed.

***Conclusions:*** Additional research studies in bariatric surgical disparities are needed. Recommendations and future directions that may help to reduce disparities in bariatric surgery are discussed.

## Introduction

Obesity (BMI ≥30) and associated comorbid medical conditions are ubiquitous in the United States. In the 2013–2014 cycle of the continuous National Health and Nutrition Examination Survey (NHANES), the weighted prevalence of obesity across race and Hispanic origin (age adjusted) overall was 37.7%.^1^ Furthermore, the overall weighted prevalence of severe obesity (BMI ≥40; class 3 obesity) across race and Hispanic origin (age adjusted) was 7.7%.

Bariatric surgery has become a frequently utilized treatment modality for obesity, type 2 diabetes mellitus (T2DM), and other components of metabolic syndrome as defined by having three or more of the following: waist circumference greater than 102 cm in men or 88 cm in women; serum triglyceride level of 150 mg/dL or greater, high-density lipoprotein level less than 40 mg/dL in men or 50 mg/dL in women; blood pressure greater than or equal to 130/85 and/or treatment with antihypertensive medication; or fasting plasma glucose level of 100 mg/dL or greater and/or treatment with medication for diabetes.^2^ Recent studies have elucidated the advantages of bariatric surgery in promoting sustained weight loss and improving comorbid medical conditions compared to lifestyle interventions and/or pharmacotherapies in isolation.^3–8^ Improvement of medical comorbidities may also persist despite regain of substantial weight following surgery.^9–11^

There is also evidence that bariatric surgery can improve mortality, likely as a result of improvements in comorbid conditions.^12^ The documented success of weight-loss surgery in normalizing glycemia has also precipitated the inclusion of bariatric procedures in guidelines and algorithms for the management of T2DM.^13,14^ According to the *American Association of Clinical Endocrinologists/American College of Endocrinology Comprehensive Clinical Practice Guidelines for Medical Care of Patients with Obesity*, consideration of bariatric surgery for weight management should be made in adult patients with BMI ≥35 kg/m^2^ and metabolic comorbid conditions, particularly if lifestyle and pharmacologic interventions have been unsuccessful.^15^

**FIG. 1.**
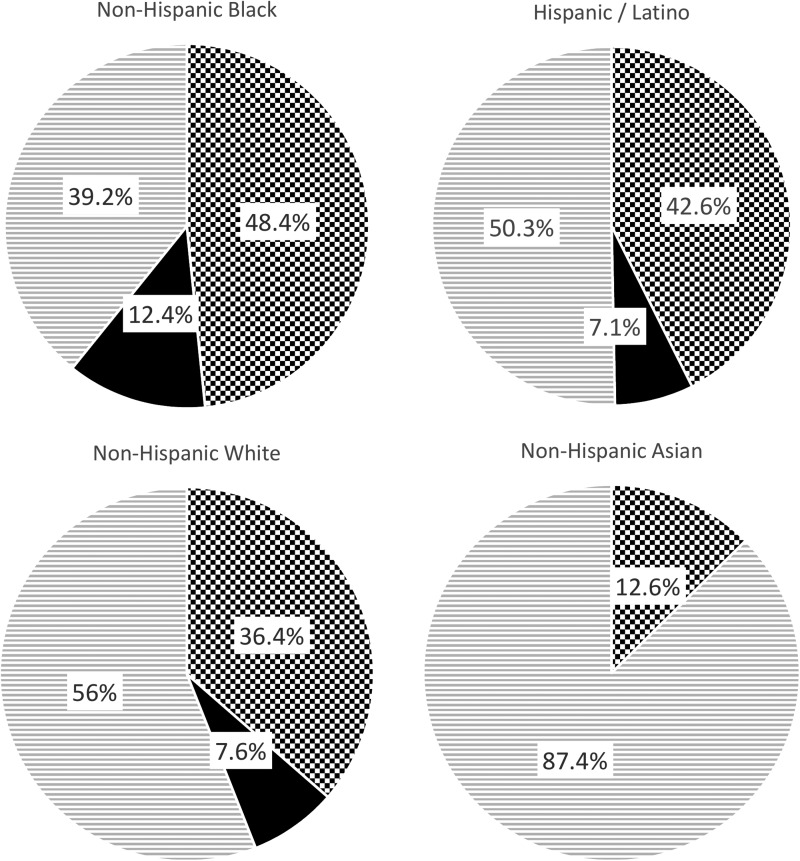
The weighted prevalence of obesity (BMI ≥30) and severe obesity (BMI ≥40) in the United States across race and Hispanic origin (age adjusted) as noted in the 2013–2014 2-year cycle of the continuous NHANES. Adapted from Flegal *et al.*^1^ NHANES, National Health and Nutrition Examination Survey. 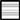 Normal Weight / Overweight (BMI <30) 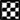 Obese (BMI ≥30) 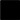 Severe Obesity (BMI ≥40)

As described, obesity and metabolic syndrome are ubiquitous in our society, affecting individuals independent of sex, age, socioeconomic status, and race/ethnicity.^1^ In the 2013–2014 cycle of the continuous NHANES, the weighted prevalence of obesity across race and Hispanic origin (age adjusted) was as follows: 48.4% among non-Hispanic blacks, 42.6% among Hispanics/Latinos, 36.4% among non-Hispanic whites, and 12.6% among non-Hispanic Asians (Fig. 1).^1^

**FIG. 2.**
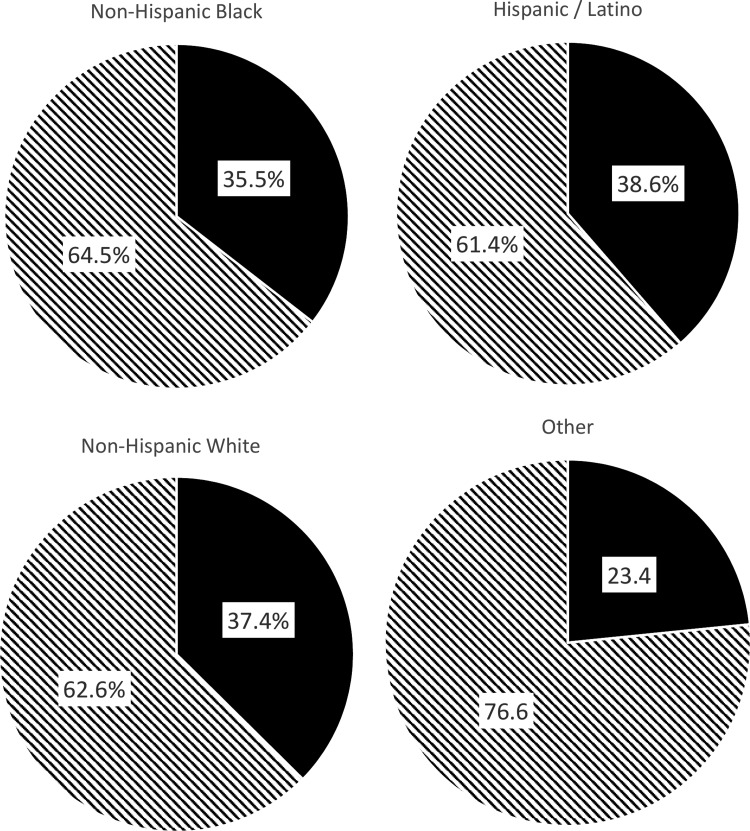
The estimated prevalence of metabolic syndrome across race and Hispanic origin. Adapted from Aguilar *et al.*^2^ 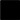 Metabolic Syndrome Does Not Meet Criteria 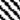 for Metabolic Syndrome

Severe obesity is also increasing in the United States. For example, between 2000 and 2010, the prevalence of a BMI >40 kg/m^2^ increased by 70%.^16^ In 2014, the weighted prevalence of severe obesity across race and Hispanic origin (age adjusted) overall was as follows: 12.4% among non-Hispanic blacks, 7.6% among non-Hispanic whites, and 7.1% among Hispanics/Latinos.^1^ Likewise, the prevalence of metabolic syndrome in the United States is substantial, with about one-third of the population meeting the criteria for the syndrome with overall higher rates among Latinos than non-Hispanic blacks or non-Hispanic white patients (Fig. 2).^2^

### Rationale

The higher prevalence of obesity and related comorbid medical conditions in traditionally underserved populations underscores the importance of equal access to indicated treatment modalities. As such, the aim of this article is to describe the disparities in seeking and accessing bariatric surgery, describe the outcomes following bariatric procedures in Latinos and African Americans, and offer recommendations and future research directions that may assist in addressing these disparities.

In the sections that follow, potential reasons why Latinos and African Americans have low rates of seeking bariatric surgery are reviewed. Disparities in access to care and financial coverage, low rates of referral by primary care providers, and cultural attitudes toward obesity in conjunction with mistrust of the healthcare system are discussed as potential contributors to the low rate of bariatric surgery in Latinos and African Americans. Disparities in bariatric surgery outcomes, comorbidities, and complications are described. Finally, future directions and recommendations that may help to reduce disparities in bariatric surgery and ultimately promote weight loss and reduction of comorbid medical conditions are discussed.

## Methods

The search engines PubMed and Ovid MEDLINE were utilized to identify original research studies and review articles published in English. These search engines were selected for their focus on healthcare issues and the ability to isolate relevant literature.

The key words used in the literature search for health inequities in bariatric surgery were as follows: African American or blacks and healthcare disparities and bariatric surgery; Latinos or Hispanic Americans and healthcare disparities and bariatric surgery. The key words used in the literature search for ethnic differences in outcomes of bariatric surgery were the same as above, along with outcomes or weight loss or T2DM or metabolic syndrome. The key words used in the literature search with regard to cultural perspectives on obesity were as follows: Latino or Hispanic American and body image; African American or black and body image. Finally, additional articles that focused on bariatric surgery and accreditation were also reviewed.

## Results

### Disparities in bariatric surgery

Several studies have described a lower rate of surgical weight-loss procedures being performed for African American/black and Latino patients compared with whites.^17–19^ For example, in one study, Martin *et al.* systematically assessed data in the NHANES to describe patients meeting eligibility criteria for bariatric surgery based on BMI and the presence of comorbid medical conditions.^19^ The authors describe the sample eligible for bariatric surgery as having a lower average household income and level of education. In addition, more than one-third of patients meeting the eligibility criteria for bariatric surgery were underinsured or uninsured. Furthermore, the eligible population was more likely to be African American or Latino than Caucasian.

Conversely, patients who underwent minimally invasive bariatric surgery were more often white, had a level of education surpassing high school, and were fully insured.^19^ Despite a clear role for bariatric surgery in the care of low-income ethnic minority populations, the issues underlying the decreased rate of bariatric surgery in certain groups are complex and multifactorial. Barriers exist at the level of access to bariatric surgery, payment and health insurance coverage, referral to surgery, and cultural beliefs surrounding medical care, all of which are reviewed herein.^1,17,18,20–35^

### Access to bariatric surgery by Latino and African American patients: payment and health insurance coverage

Insurance coverage and payments appear to be related to some of the disparities in weight-loss surgery for Latinos and African Americans. However, bariatric surgical procedures are not only a safe and effective tool for the management of obesity and associated comorbidities but have also been proven to be cost-effective.^36^

Accreditation of bariatric surgery facilities started in 2004 with the American Society for Metabolic and Bariatric Surgery (ASMBS) and in 2005 with the American College of Surgeons (ACS).^37^ In 2006, the Centers for Medicare and Medicaid Services (CMS) instituted a restriction of coverage of weight-loss surgery to sites accredited as Centers of Excellence (COE).^38^ The goal of the COE Program was to designate programs with outstanding surgical outcomes with a low rate of complications.^31^ Later, in 2012, the ACS and the ASMBS combined their respective accreditation programs to create the Metabolic and Bariatric Surgery Accreditation and Quality Improvement Program (MBSAQIP).^39^

One of the primary concerns that was discussed in the literature following the initial restriction of the CMS insurance payment coverage to COE-accredited programs was the potential for decreased access to bariatric procedures by certain ethnic minority groups such as African Americans/blacks and Hispanics/Latinos.^19,40^ For example, Nicholas and Dimick reported that there was a decline in the proportion of non-white Medicare patients undergoing bariatric surgery at 429 hospitals following the CMS restriction of care to COEs.^40^

On the contrary, another study described an increase in the proportion of Medicare patients receiving surgery at COEs with a specific increase in Medicare patients who were black, between 2008 and 2011, from two high-volume surgical sites in New York and Florida.^26^ However, this same study also reported that Medicare patients traveled further for access to the procedures than non-Medicare patients at these particular sites.^26^ A difference in findings from these two studies could likely be explained by variations in the incidence of surgery among ethnic minorities by geographic location as the former study assessed patients from 429 hospitals, whereas the latter only analyzed two sites.

In 2013, the CMS abandoned the restriction of reimbursement to COE programs after an analysis failed to demonstrate that recognized centers achieved better outcomes than nonaccredited institutions.^41^ Although Medicare reimbursement is no longer restricted to accredited institutions, substantial data indicate that bariatric facility accreditation is associated with improved outcomes.^37,42^ As such, in a recent position statement, ASMBS recommended that all facilities participating in bariatric surgery in the United States enroll in an accreditation program and that patients strongly consider only seeking care at such recognized centers to best ensure quality of care.^43^

Currently, with the Patient Protection and Affordable Care Act (ACA), plans offered through the state ACA exchanges, as well as private insurers, are required to cover screening and counseling for obesity for children and adults.^44^ However, coverage for bariatric surgery is not required for private insurers or state-run exchanges.^44^ In terms of adolescents, as assessed in *The National Poll on Children's Health*, public opinion regarding Medicaid coverage for obesity is supportive of medical management but not in regard to surgical treatment strategies.

In a study using data from the National Inpatient Sample database from 2003 to 2010, the authors noted that significantly more bariatric procedures were performed in the northeastern states compared with those states that make up the so-called Diabetes Belt.^45^ The Diabetes Belt consists of 644 counties in 15 states (Alabama, Arkansas, Florida, Georgia, Kentucky, Louisiana, Mississippi, North Carolina, Ohio, Pennsylvania, South Carolina, Tennessee, Texas, Virginia, and West Virginia) whose residents are more likely to have T2DM in comparison to the rest of the country.^46^ This region of the country also has a poverty rate of about 21% (compared with 14% in nondiabetes belt states) and 24% of the population is African American or black (relative to 9% in other states).^46,47^ Finally, even for patients for whom Medicare and Medicaid coverage of bariatric surgery is adequate, rates of bariatric surgery remain lower compared with those with private insurance.^48^

Overall, it is imperative that individuals who are obese are provided with information about nonsurgical and surgical treatment options for obesity. In addition, it is important that individuals who are obese are also being referred by healthcare providers to surgical centers to pursue treatment options for obesity, of which bariatric surgery may be an option for some individuals. Below we review apparent disparities in referrals and identify other potential reasons that ethnic minorities may pursue bariatric surgery at lower rates than whites.

### Referral to and decision to pursue bariatric surgery

A contributing factor to the lower rate of bariatric surgery in ethnic minorities likely concerns the identification of eligible patients and their subsequent referral to a bariatric program for consideration of surgery. Introduction to bariatric surgery as a treatment modality may initially occur via primary care providers and medical specialists who manage obesity-related comorbidities. Unfortunately, studies have found that eligible ethnic minority patients are overall less likely to undergo bariatric surgery for management of obesity than white patients, and that referral rates may be lower for this population.^28,33,35^

Bariatric surgery patients are most often middle-aged Caucasian females despite increased rates of obesity in men and women from ethnic minority groups.^28^ However, results from one study indicated that ethnic minorities who are referred to and proceed with an initial surgical evaluation are just as likely to proceed with surgery as their Caucasian peers.^49^

It is worth noting that disparities in seeking surgical treatment for obesity are not apparent at all bariatric surgery centers. For example, in one study that we conducted at our academic medical center, we examined excess weight loss postbariatric surgery across racial/ethnic groups from 2008 to 2014 and the sample included a large number of ethnic minorities (blacks [*n* = 360], Latinos [*n* = 200], whites [*n* = 141], others [*n* = 48]).^50^ One reason for this may be because our institution is located in a city with a large population of blacks and Latinos.^51^ Differences in the types of insurances that institutions accept (e.g., public vs. private insurances) may also be related to the decision to seek surgery at one medical center/hospital versus another. Our bariatric surgery center also offers language interpreter services, as well as has providers who speak Spanish, which may also be attractive to some patients seeking services in their preferred language.

The reasons for the lower rate of bariatric surgery procedures in eligible ethnic minorities versus Caucasians have not been systematically examined, but possible reasons may include that ethnic minority patients are either not being referred as frequently for bariatric surgery, or are themselves not seeking or choosing to pursue bariatric surgical procedures. Moreover, in addition to disparities in health insurance, both poor access to primary and specialty care and possible mistrust of the healthcare system are also purportedly related to the decreased rates of referral to and lower rates of bariatric surgery procedures in certain populations.^52–54^

Indeed, medical system mistrust has been shown to be disproportionately prevalent in ethnic minority populations.^55–58^ There is also evidence that such mistrust contributes to decreased use of primary care and increased rates of obtaining care from emergency services.^55^ As primary care serves as an entry point by which obese patients can obtain surgical care, it is possible that medical mistrust may interfere with the process of seeking bariatric procedures.

Despite the previously described inclusion of bariatric surgery in the treatment algorithms for both obesity and T2DM,^14,15^ data suggest that many primary care physicians are not routinely referring eligible patients for consideration for weight-loss procedures.^34^

In one study of obese Latino patients with BMI >35 kg/m^2^, the authors reported that only 22% of patients noted that bariatric surgery was recommended by their physician, although a majority (66%) reported that they would consider weight-loss surgical procedures if suggested.^34^ In that same study, about half of African American/black obese patients were provided with information about weight-loss surgery from their physician, while only 20% of physicians *recommended* bariatric surgery to eligible individuals.^34^ Results from this study also revealed that both Latino and African American/black patients reported that they would be more likely than Caucasian patients to consider surgery if it was recommended by their physician. These findings illustrate the important role of physicians in the care of obese patients.

One possible reason for the low referral rate of eligible patients may be due to the need for additional guidance about how to talk to patients about bariatric surgery, and an incomplete understanding of the risks and benefits of bariatric surgery by both clinicians and patients, both of which are areas in need of future investigation.

To further examine disparities in referrals and decisions to proceed with bariatric surgery, these issues are explored below as it relates specifically to Latinos and African Americans. Weight loss and metabolic outcomes following bariatric surgery in ethnic minorities are also reviewed in the following sections.

### Hispanics/Latinos and rates of bariatric surgery

In 2015, it was estimated that the Hispanic/Latino population comprised 17% of the total U.S. population, and this percentage is projected to increase in the future.^25,26^ With known high rates of obesity, Hispanics/Latinos would undoubtedly greatly benefit from the management of obesity through medical, surgical, nutritional, and psychological approaches. Furthermore, about one-third of Latinos have at least one risk factor for cardiovascular disease.^59^

Despite a clear role for weight-loss interventions, in one study, Hispanics/Latinos were less likely than Caucasians to receive laparoscopic gastric bypass surgery in the United States between 2002 and 2008.^35^ Furthermore, while the proportion of African Americans/blacks receiving gastric bypass surgery increased during this time period, this is not true of Hispanics/Latinos.^35^ Moreover, the rate of bariatric surgery is particularly small in Hispanics/Latinos from the lowest socioeconomic quintiles.^18^ Therefore, it is important to examine possible reasons why Hispanics/Latinos may not be receiving bariatric surgery at the same rate as other ethnic groups, given their high rates of obesity.

### Cultural attitudes about obesity and weight in Hispanics/Latinos

Cultural attitudes concerning overweight and obesity may contribute to decreased efforts to manage or lose weight.^60–65^ A number of studies have explored cultural attitudes concerning obesity and weight (or thinness) in Hispanic/Latino populations.^64^ Several studies have revealed that perceptions of being overweight are more common in white women compared with both African Americans/blacks and Latinas.^63,64^ Weitzman *et al.* utilized focus groups to assess motivations for weight loss in Latina patients with T2DM and noted less emphasis on a desire to reduce body size and greater interest in improvement in skin health, energy levels, and reduction in unwanted hair growth.^65^

Overall, it is important to develop culturally sensitive methods of counseling patients regarding obesity and overweight and to, in turn, develop culturally appropriate methods of disseminating information about weight-loss interventions, particularly for invasive procedures.^62^ For Latinos, referral to bariatric surgery and recommendations regarding weight loss may be more effective if practitioners emphasize markers of health independent of body weight and BMI. In addition, counseling may differ for Latinos who are more integrated into American culture, as one study noted that these patients more often have an understanding that overweight and obesity are detrimental to their health compared with Latinos living in Mexico.^61^ As information about the health risks associated with overweight and obesity increases, it may be that Latinos will more likely seek both surgical and nonsurgical treatment of obesity.

### African Americans or blacks and rates of bariatric surgery

As of 2015, African Americans/blacks comprised an estimated 13% of the population in the United States, a population disproportionately affected by obesity, as previously detailed.^25^ Despite increasing rates of referral for bariatric surgery among African Americans or blacks, the absolute proportion of patients who undergo weight-loss procedures remains low.^32,35^ Black males in particular undergo fewer gastric bypass procedures relative to black females, and white and Hispanic patients.^28,32,34^ Moreover, despite increasing rates of coverage by Medicaid programs, one study examining bariatric surgery procedures between 1999 and 2010 implicated poor access to private health insurance as a primary factor driving the lower incidence of bariatric surgery in African American/black patients.^27^

Similar to Latinos, it is apparent that African Americans/blacks are not pursuing weight-loss surgeries at a rate that is proportionate to the prevalence of obesity and comorbid medical conditions in this population.^27–35^ While many of the reasons for these disparities are similar to those of Latinos, there are also circumstances unique to African Americans/blacks that may be related to the decreased rates of bariatric surgery.

### Cultural attitudes about obesity and weight in African Americans or blacks

Similar to Hispanics/Latinos, cultural attitudes about overweight and obesity may contribute to decreased efforts to manage or lose weight among African Americans/blacks.^60–65^ Several studies have found that black women, in particular, exhibit a preference for overweight and obese body sizes^64,66,67^ and black men more frequently desire to date women who are overweight or obese than normal weight women.^64,66–68^ Furthermore, in one study, black men and women exhibited a significant misperception of their weight and frequently underestimated their BMI or waist circumference.^69^ Moreover, in another study, obese and overweight African American/black girls tended to select larger ideal body image figures, which were not within the normal weight range, compared with their normal weight peers.^70^

The preference for an overweight and obese body size among African Americans/blacks may also be related to lower rates of pursuing surgical weight-loss procedures. However, this relationship has not been examined specifically in African Americans/blacks.

### Bariatric surgery outcomes in African Americans or blacks and Hispanics/Latinos

Bariatric surgery is associated with a loss of excess body weight, ranging from about 40–75%, depending on the procedure.^21^ In conjunction with the aforementioned improvements in comorbid medical conditions, weight-loss procedures can lead to significant improvements in the quality of life of patients who are obese.^8,71,72^ However, as seen in the section below, outcomes appear to vary across certain ethnic and racial groups.

### Weight loss in African Americans or blacks and Hispanics/Latinos

Studies vary with respect to differences in postsurgical excess weight loss across ethnic/racial groups. For example, numerous studies have reported significantly greater excess weight loss at certain time points following surgery in whites relative to African Americans/blacks and Latinos.^17,20,22,23,25,29,30,50^ For example, a recent study demonstrated that whites had a greater percent excess weight loss (%EWL) than blacks at 6 months following surgery, although not at 12, 24, and 36 months and that Hispanics had a greater %EWL than blacks at all follow-up time points.^50^

In the largest retrospective review of more than 2000 Latinos undergoing bariatric surgery, although excess weight loss was significant, it was less in Hispanics compared with results from a smaller non-Hispanic white cohort from the same institution and time period.^73^ An additional study found no absolute difference in the proportion of African American/black, Hispanic, and white women exhibiting significant weight loss after surgery, but did demonstrate greater weight loss in African American or black men compared with white men.^24^ By contrast, other studies have failed to demonstrate a difference in weight loss across racial/ethnic groups.^32^

It is not clear whether the factors contributing to the difference in weight-loss outcomes across ethnic/racial groups is a result of modifiable or nonmodifiable risk factors. In one study, results from a regression analysis revealed that older age and higher preoperative BMI predicted less postoperative weight loss and that greater presurgical weight loss predicted greater postsurgical weight loss.^74^ The same study also found that racial differences in weight loss persisted even after controlling for certain psychosocial and demographic factors.

An additional study documented that a preoperative diagnosis of T2DM predicted successful weight loss following surgery in African American/black women.^30^ Finally, in one study, dietary intake after surgery did not appear to be associated with postoperative weight loss.^75^ However, additional research is needed to examine dietary intake across ethnic/racial groups pre- and postsurgery.

### Improvement in comorbid medical conditions following bariatric surgery

Numerous studies have found a lower excess weight loss in African American/black and Latino patients relative to white patients, although there are discrepancies across some studies with regard to when these differences in weight-loss outcomes appear and whether or not they apply to both African Americans and Latinos.^17,20,22,23,25,29,30,50^

Despite a lower degree of excess weight loss in African American/black and Latino patients compared with white patients, a majority of studies have failed to demonstrate significant differences between African Americans/blacks and whites in the resolution or improvement of certain comorbid medical conditions postbariatric surgery.^29,32,76,77^ For example, Araia found that the rate of resolution of T2DM postbariatric surgery in a predominantly African American/black population was similar to that reported in mostly white samples.^76^

Despite most studies suggesting no difference in the improvement of comorbid conditions postoperatively, a few studies have demonstrated differences in such outcomes. For example, while Sudan *et al.* documented decreased prevalence of multiple medical comorbidities 1 year following surgery across all ethnic groups, blacks demonstrated higher rates of diabetes, hypertension, and congestive heart failure postbariatric surgery compared with whites after controlling for potential confounders.^32^ Coleman *et al.* also demonstrated that although black and Latino patients exhibited significant improvements in metabolic syndrome following bariatric surgery, those groups exhibited lower rates of improvement relative to whites.^77^

### Complications following bariatric surgery

Bariatric surgery is associated with a very low rate of severe complications and mortality.^8^ Despite this finding, studies have noted that both serious and minor adverse events may occur at a higher rate in black patients relative to other ethnic groups.^32^ Moreover, mortality rates may be higher among African Americans or blacks compared with white patients.^78^

While Turner *et al.* did not observe a difference in mortality across racial/ethnic groups, they did find that certain serious complications occurred more frequently in black (pulmonary embolism) and Latino (acute renal failure requiring dialysis; need for blood transfusion) patients postbariatric surgery compared with white patients.^79^ It is not yet clear if the increased incidence of adverse events in certain ethnic/racial groups is a result of genetic, metabolic, or psychosocial factors.

## Discussion

Bariatric surgery is a valuable tool for the treatment of severe obesity and associated medical comorbidities. However, despite the prevalence of obesity and metabolic syndrome in ethnic/racial minority populations, Latinos and blacks undergo bariatric surgery procedures less frequently than whites.

As reviewed herein, disparities in rates of bariatric surgery across ethnic groups may exist at the level of the healthcare provider (e.g., referral for surgery) and with respect to insurance coverage and payments. Furthermore, disparities in rates of bariatric surgery are also likely due to variability in cultural perspectives of preferred body size, attitudes toward obesity, and possible mistrust of the healthcare system. Together, these are possible factors that may contribute to the lower rate of bariatric surgery in African Americans/blacks and Latinos compared with whites.

### Recommendations and future directions

Several recommendations are outlined below that may help to address the disparities in bariatric surgery in Latinos and African Americans (Table 1). First, healthcare providers in both community and clinic/hospital settings may benefit from receiving additional information about bariatric surgery. It is essential that accurate information is provided about the benefits, risks, and outcomes of surgical and nonsurgical approaches to overweight and obesity, particularly for healthcare providers who provide primary medical care to multiethnic populations. Stereotypes about obesity and bariatric surgery may also affect the way in which healthcare providers make recommendations for weight loss.

**Table 1.** Suggestions for Addressing Disparities in Bariatric Surgery

*Reported disparities in the literature*	*Suggestions for future studies/knowledge gap*
Rates of bariatric surgery are disproportionately low in African American and Latino qualifying patients relative to white patients.^17–19,28,33,35^	Systematic study to determine the particular multifactorial reasons for the low rates of referral for bariatric surgery.
Eligible ethnic minority patients are not consistently being referred for bariatric surgery by primary care providers.^34^	Investigate the reasons for low referral rates by conducting a study within offices of primary providers who work with underserved and ethnically/racially diverse populations.
African Americans and Latinos have fewer negative associations with overweight and obese, relative to whites.^63–65^	Design educational and informational tools for providers to encourage culturally sensitive communications with patients who may be eligible for bariatric surgery.
Studies suggest a lower percentage of excess weight loss after bariatric surgery in Latinos and African Americans compared to whites.^17,20,22,23,25,30,32,50^	Translational research studies should be conducted, which may contribute to understanding the physiological, genetic, sociological, and psychological factors that may be related to lower weight loss in ethnic minorities.
There is some evidence to suggest that African American and Latino patients demonstrate lower rates of improvement of medical comorbidities following bariatric surgery, relative to white patients.^32,77^	As above, translational research studies should be conducted to investigate the mechanisms that may underlie the multiple factors contributing to disparities in comorbid medical conditions postbariatric surgery.

Specific information about the multiple factors related to overweight/obesity, as well as guidance about how to speak to patients about bariatric surgery in general should also be a part of this information. Sensitivity training as it relates to the care of overweight and obese patients may be helpful in this regard. Such trainings have been used in hospitals and healthcare systems.^80^

Future research is also needed to understand the ways in which weight-loss counseling in general may be tailored to the particular cultural beliefs and needs of ethnic minority patients. In addition, data about the race/ethnicity of surgeons and patients may provide information about whether referrals, rates of bariatric surgery, and outcomes differ as a function of the race/ethnicity of surgeons and their patients.

Second, disparities in outcomes following bariatric surgery for ethnic minorities are concerning and warrant further examination by researchers and healthcare providers who develop treatment plans for obese African American/black and Latino patients. It is recommended that additional research is conducted to further explore the reasons for ethnic/racial variability in weight loss and improvement of medical comorbidities such as metabolic syndrome following surgery so that culturally appropriate postoperative programs can be developed. Future research should also examine additional psychosocial, metabolic, and genetic predictors of weight-loss success in ethnic minority groups following surgery.^30^

Third, additional research is needed to investigate reasons for the mistrust of the medical system that has been reported in certain studies and whether this applies to the surgical treatment of obesity. It is further recommended that patient advocates, patient navigators, and community members be involved in the development of novel ways to enhance healthcare delivery as it relates to bariatric care and obesity management in general.

Finally, it is imperative that information be provided to Latinos and African Americans in clinical and nonclinical (community) settings about the health risks associated with higher BMI levels and large waist circumferences, as well as information about the safety, effectiveness, and procedures involved in bariatric surgery. This information should be provided in both English and Spanish to overcome barriers to treatment seeking as it relates to accessing information in the preferred language of patients.

As Latinos and African Americans develop a better understanding of the health risks associated with obesity and increased knowledge about treatment options for obesity, it is possible that they will seek both nonsurgical (medical, psychological, nutritional, etc.) and surgical approaches to weight loss, which may ultimately help to reduce rates of obesity.

### Notes

The words “Hispanics” and “Latinos” as well as “African Americans” and “blacks” will be used interchangeably in this article, depending on the study reviewed.
